# Cervical fractures with associated spinal cord injury in children and adolescents: epidemiology, costs, and in-hospital mortality rates in 4418 patients

**DOI:** 10.1007/s11832-015-0657-9

**Published:** 2015-05-08

**Authors:** Amit Jain, Jaysson T. Brooks, Sandesh S. Rao, Michael C. Ain, Paul D. Sponseller

**Affiliations:** Department of Orthopaedic Surgery, The Johns Hopkins University School of Medicine, 601 N. Caroline Street, Baltimore, MD 21287 USA

**Keywords:** Cervical spine, Children, Death, Spinal cord injury, Trauma

## Abstract

**Background:**

Cervical spine fractures with spinal cord injury (CFSCI) can be devastating. We describe the epidemiology of children and adolescents with CFSCI.

**Methods:**

Using the Nationwide Inpatient Sample (NIS) database, we identified 4418 patients (≤18 years old) who had CFSCI from 2000 through 2010. Outcomes of interest were patient characteristics (age, sex), injury characteristics [fracture location, spinal cord injury (SCI) pattern], economic variables (duration of hospital stay, total hospital charges), and mortality.

**Results:**

Upper cervical fractures (UCFs) occurred half as often (31.4 %) as lower cervical fractures (LCFs; 68.8 %). Among patients <8 years old, 73.6 % had UCFs; among patients ≥8 years old, 72.3 % had LCFs. Overall, 68.7 % had incomplete SCI, 22.4 % had complete SCI, 6.6 % had central cord syndrome, and 2.3 % had anterior cord syndrome. Patients with complete SCI had the longest hospital stays and highest hospital charges. The overall in-hospital mortality rate was 7.3 %, with a sixfold higher rate in patients <8 (30.6 %) vs. those ≥8 (5.1 %) years old (*p *< 0.001). There was a threefold higher mortality rate in patients with upper (13.5 %) vs. lower (4.3 %) cervical fractures (*p* < 0.001). Patients with complete SCI had a 1.85-fold higher mortality rate than patients with other cord syndromes (*p* < 0.001).

**Conclusions:**

Patients <8 years old were more likely than older patients to sustain UCFs. Patients with UCFs had a significantly higher mortality rate than those with LCFs. Patients with complete SCI had the longest duration of hospital stay and highest hospital charges and in-hospital mortality rate.

## Introduction

Spinal cord injuries in children have devastating consequences. The overall reported incidence of pediatric spinal injuries is 2 per 100,000 in the United States, and 90 % of all children who experience a spinal cord injury are >15 years old [1]. Cervical spine injuries have been reported to comprise 60–80 % of all pediatric spine injuries, in contrast to 30–40 % in adults [2].

It has been postulated that biomechanical and anatomical features unique to the immature pediatric spine may account for different injury patterns in children compared with those in adults. In young children, head size is large in proportion to body size, causing the upper cervical spine to be subject to greater force than the lower cervical spine, thus making the upper cervical spine more prone to injury. To our knowledge, the largest epidemiologic study of pediatric cervical trauma was by Patel et al. [3], who analyzed 1098 children in the National Pediatric Trauma Registry from 1988 to 1998. The authors found that children had upper cervical spine injuries nearly twice as frequently as lower cervical spine injuries, and that they presented in a bimodal distribution at ages 3 and 16 years [3]. A recent study of 540 children with cervical spine injuries found that children <7 years old were more commonly injured in the occiput–C2 region, whereas children 8–15 years old were more commonly injured in the subaxial cervical region (C3–C7); 21 % of the patients in their series had associated neurologic deficits [4].

Our goals were to describe the epidemiology of children and adolescents with cervical spine fractures and associated spinal cord injury (CFSCI) and to analyze duration of hospital stay, total hospital charges, and in-hospital mortality rates. We hypothesized that patients with upper cervical fractures (UCFs) and those with complete cord injuries would have the highest mortality rates.

## Materials and methods

### Data source

We used the Nationwide Inpatient Sample (NIS) database, maintained by the United States Agency for Healthcare Research and Quality (Rockville, Maryland), for our study. The NIS is the largest all-payer inpatient care database and contains a representative 20 % sample of all inpatient admissions, which can be converted to national estimates using discharge weights provided within the database. Use of this publicly available database is exempt from institutional review board approval.

### Query method

The database was searched from 2000 through 2010 to identify all patients ≤18 years old who had CFSCI [using the International Classification of Diseases, 9th Revision, Clinical Modification [5] (ICD-9-CM) diagnostic code 806.0 and its variations]. The discharge weights provided were used to create national estimates, and 4418 patients were estimated to have CFSCI. Using the national estimates, information on the following outcomes of interest was tabulated: patient characteristics (age, sex), injury characteristics (fracture location and cord injury pattern), economic variables (duration of acute hospital stay, not including transition care and rehabilitation facility stay, and total hospital charges), and death. Fractures were classified on the basis of location as UCFs, defined as fractures involving the C1–C4 vertebrae (ICD-9-CM codes 806.00–806.04), or as lower cervical fractures (LCFs), defined as fractures involving the C5–C7 vertebrae (ICD-9-CM codes 806.05–806.09). Fractures were classified on the basis of the following cord injury patterns: complete cord injury (ICD-9-CM codes 806.01 and 806.06), incomplete cord injury (codes 806.00, 806.04, 806.05, and 806.09), anterior cord syndrome (codes 806.02 and 806.07), and central cord syndrome (codes 806.03 and 806.08).

### Statistical methods

Stratified sampling weights provided with the NIS database were used to calculate national estimates. The *t*-test was used to assess the differences in patient age, duration of hospital stay, and total hospital charges by fracture location. The *t*-test was also used to assess the differences in duration of hospital stay and total hospital changes by patient sex and the differences in patient age by mortality. Analysis of variation was used to assess the variance in patient age, duration of hospital stay, and total hospital charges by cord syndrome. The Chi-square test was used to assess (1) the differences in fracture location by patient sex; (2) the relationship between cord syndrome and patient sex; and (3) the differences in mortality rate by patient sex, fracture location, and cord syndrome. Simple linear regression models were used to assess the relationships among patient age, duration of hospital stay, and total hospital charges. Statistical significance was set at *p* < 0.05 for all analyses.

## Results

### Patient characteristics

**Fig. 1:**
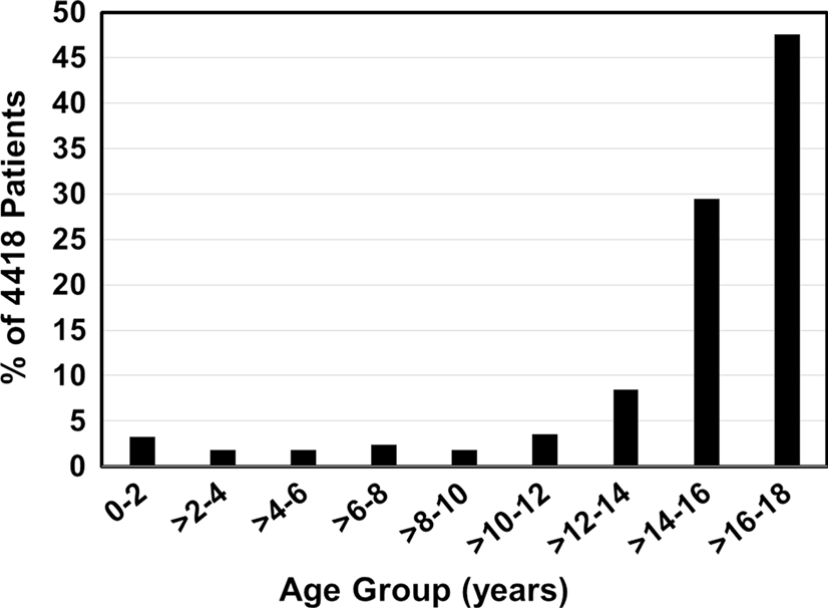
Age distribution in 4418 patients with cervical spine fractures with spinal cord injury. National Inpatient Sample, 2000–2010

The mean (standard deviation, SD) age at injury was 15.1 (3.9) years, and most patients were in the 16–18 years age group (Fig. 1). Of the 4418 patients, 71.4 % were boys and 28.6 % were girls. Of those <8 years old (352 patients), 55.5 % were boys and 44.5 % were girls. Of those ≥8 years old (4066 patients), 72.8 % were boys and 27.2 % were girls.

### Injury patterns

**Fig. 2:**
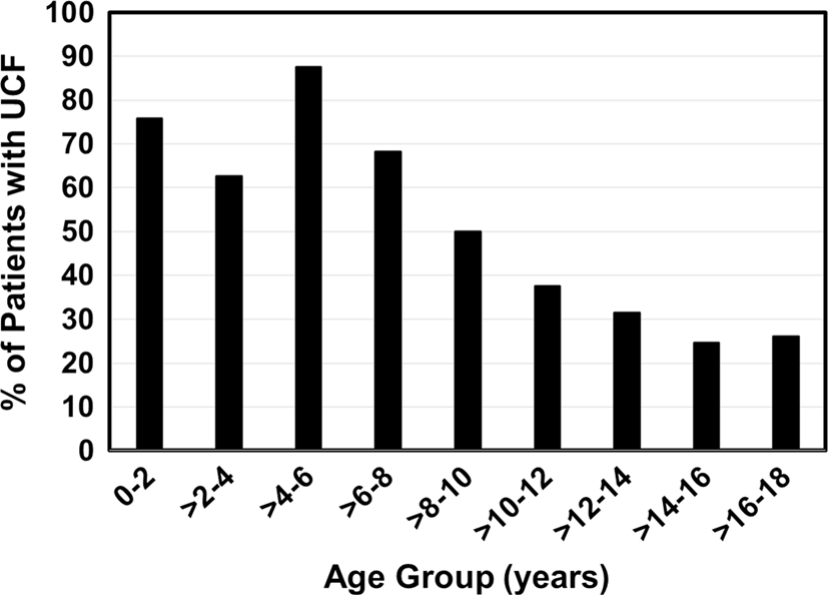
Upper cervical fractures (UCFs) are more common in patients <8 years old. At ≥8 years old, lower cervical fractures become more common. National Inpatient Sample, 2000–2010

Of the 4418 patients, 31.4 % had a UCF and 68.6 % had an LCF. The mean (SD) age was significantly lower in patients with UCF than in those with LCF [13.4 (5.3) years vs. 15.9 (2.9) years, respectively; *p* < 0.001]. In patients <8 years old, 73.6 % had a UCF; in patients ≥8 years old, 72.3 % had an LCF (Fig. 2). There was no significant difference in the rates of UCF and LCF by sex (*p* = 0.132).

Most patients (68.7 %) had incomplete spinal cord injury; 22.4 % patients had complete spinal cord injury. There was no significant variation in patient age (*p *= 0.734) or sex (*p* = 0.627) by cord syndrome.

### Duration of hospital stay and total hospital charges

The mean (SD) duration of hospital stay for patients with CFSCI was 17.3 (21.6) days. There was no significant relationship between the duration of hospital stay and patient age (*p* = 0.145) or sex (*p* = 0.134). There was no significant difference in the duration of stay between patients with UCF or LCF (*p* = 0.811). There was a significant variation in the duration of stay by cord syndrome (*p* < 0.001); patients with complete cord injury had the longest average hospitalization (Table 1).

**Table 1:** Mean duration of hospital stay, total hospital charges, and mortality rate by spinal cord syndrome in 4418 children and adolescents. National Inpatient Sample, 2000–2010

Syndrome	Percentage of patients	Mean (SD) duration of hospital stay, days	Mean (SD) total hospital charges, $	Mortality rate, %
Complete cord injury	22.4	23 (24)	207,097 (187,300)	11.1
Incomplete cord injury	68.7	16 (21)	137,608 (161,105)	6.9
Anterior cord syndrome	2.3	10 (9)	94,536 (76,902)	0
Central cord syndrome	6.6	12 (22)	67,441 (56,838)	1.8

The mean (SD) total hospital charge for patients with CFSCI was $147,523 ($165,319). There was no significant relationship between total charges and patient age (*p* = 0.599) or sex (*p* = 0.388). There was no significant difference in the total charges between patients with UCF and those with LCF (*p* = 0.944). There was significant variation in the total charges by cord syndrome (*p* < 0.001); patients with complete cord injury had the highest total charges, and those with central cord syndrome had the lowest (Table 1).

### Mortality and associated factors

**Fig. 3:**
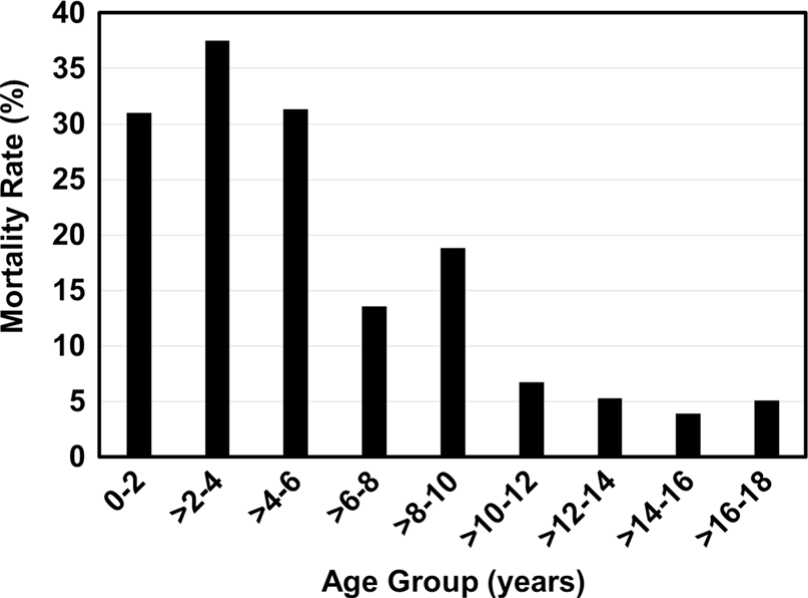
In-hospital mortality rates among 4418 patients with cervical spine fractures with spinal cord injury by age group. National Inpatient Sample, 2000–2010

The overall in-hospital mortality rate associated with pediatric CFSCI was 7.3 % (Fig. 3). Patients who died were significantly younger than those who did not [mean (SD), 11.4 (6.5) vs. 15.4 (3.6) years, respectively; *p* < 0.001]. There was a sixfold higher mortality rate in patients <8 years old vs. those ≥8 years old (30.6 vs. 5.1 %, respectively; *p* < 0.001). There were no differences in the mortality rate by sex (*p *= 0.650).

The associated mortality rates for UCFs and LCFs were 13.5 and 4.3 %, respectively (*p* < 0.001). There was a significant difference in the mortality rate by cord syndrome (*p* = 0.031); patients with complete cord injury had a 1.85-fold higher mortality rate compared with that of the other three groups combined (*p* < 0.001).

## Discussion

We found that patients <8 years old predominantly had UCFs, whereas patients ≥8 years old tended to sustain LCFs. This finding is consistent with those in the literature [2, 6–10]. This phenomenon may be attributed to the age-dependent hypermobility of the pediatric spine, which starts to take on adult characteristics at approximately 8 years of age [8–12]. The spine in these younger children has intrinsically less stability compared with that in adolescents because of weaker neck muscles, incomplete vertebral ossification, more horizontally orientated articulation of the facet joints, and ligamentous laxity. These factors, in addition to larger head size (which creates a fulcrum of cervical motion much higher than that in adolescents and adults), make upper cervical injuries substantially more likely in younger children [11, 13].

In our study, boys had CFSCI more commonly than girls. This finding is consistent with those in other series in the literature, in which boys were reported to outnumber girls in cervical spine trauma [3, 6, 7, 9].

Cervical spine fractures with associated spinal cord injury can have a high associated mortality rate. In our study, the overall mortality rate was 7.3 % and the mortality rate was approximately threefold higher in patients with UCFs than in those with LCFs. In addition, we found that patients <8 years old had a sixfold higher mortality rate than those ≥8 years old. The high mortality rate in younger children is likely the result of associated head injury and polytrauma. This finding is supported by the report of Orenstein et al. [12], who found a 26 % death rate in patients <8 years old and concluded that death in younger patients was not caused exclusively by the higher level of cervical injury, but that it occurred more often in the presence of concomitant head injury and polytrauma. We also found that patients with complete cord injuries had a 1.9-fold higher mortality rate compared with those with other cord syndromes. Patients with complete cord injuries likely sustained high-energy injuries and, perhaps, were more likely to have concomitant head injury and polytrauma.

Our study had a few limitations. The NIS is designed to collect discharge information from inpatient hospital admissions. As a result, it captures only cross-sectional data, and we are not able to follow patients longitudinally and determine postdischarge mortality rates. In addition, because the NIS relies on ICD-9-CM codes, we were limited in our ability to distinguish between fracture types in terms of their clinically relevant features. In addition, we were not able to study the mechanism of injury or the clinical course of these patients during their hospital stays. The mechanism of injury is a particularly important consideration and may explain the difference in injury patterns by age and sex that we noted in our analysis. Unfortunately, we were not able to analyze this as a variable using the NIS. Despite these shortcomings, ours is the largest series on pediatric cervical fractures with associated cord injury. The largest previous study on the epidemiology of pediatric spinal injuries was performed by Patel et al. [3] using the 1988–1998 National Pediatric Trauma Registry. However, only 17 % of children in their series had CFSCI, and the patient characteristics of that subgroup were not reported.

## Conclusions

Patients <8 years old were more likely to sustain a UCF, and patients ≥8 years old were more likely to sustain an LCF, which may be a result of biomechanical differences with age that emerge during normal growth. Patients with UCFs had a threefold higher rate of death than those with LCFs. There was a significant difference in patient outcomes by cord syndrome, and patients with complete cord injury had the longest duration of hospital stay and highest total hospital charges and in-hospital mortality rate. An understanding of these factors is important for teams treating these children and adolescents.
